# Age and Racial/Ethnic Disparities in Prepregnancy Smoking Among Women Who Delivered Live Births

**Published:** 2011-10-15

**Authors:** Van T. Tong, Patricia M. Dietz, Lucinda J. England, Sherry L. Farr, Shin Y. Kim, Denise D'Angelo, Jennifer M. Bombard

**Affiliations:** Division of Reproductive Health, National Center for Chronic Disease Prevention and Health Promotion, Centers for Disease Control and Prevention; Division of Reproductive Health, National Center for Chronic Disease Prevention and Health Promotion, Centers for Disease Control and Prevention, Atlanta, Georgia; Division of Reproductive Health, National Center for Chronic Disease Prevention and Health Promotion, Centers for Disease Control and Prevention, Atlanta, Georgia; Division of Reproductive Health, National Center for Chronic Disease Prevention and Health Promotion, Centers for Disease Control and Prevention, Atlanta, Georgia; Division of Reproductive Health, National Center for Chronic Disease Prevention and Health Promotion, Centers for Disease Control and Prevention, Atlanta, Georgia; Division of Reproductive Health, National Center for Chronic Disease Prevention and Health Promotion, Centers for Disease Control and Prevention, Atlanta, Georgia; Division of Reproductive Health, National Center for Chronic Disease Prevention and Health Promotion, Centers for Disease Control and Prevention, Atlanta, Georgia

## Abstract

**Introduction:**

Prenatal smoking prevalence remains high in the United States. To reduce prenatal smoking prevalence, efforts should focus on delivering evidence-based cessation interventions to women who are most likely to smoke before pregnancy. Our objective was to identify groups with the highest prepregnancy smoking prevalence by age within 6 racial/ethnic groups.

**Methods:**

We analyzed data from 186,064 women with a recent live birth from 32 states and New York City from the 2004-2008 Pregnancy Risk Assessment Monitoring System (PRAMS), a population-based survey of postpartum women. We calculated self-reported smoking prevalence during the 3 months before pregnancy for 6 maternal racial/ethnic groups by maternal age (18-24 y or ≥25 y). For each racial/ethnic group, we modeled the probability of smoking by age, adjusting for education, Medicaid enrollment, parity, pregnancy intention, state of residence, and year of birth.

**Results:**

Younger women had higher prepregnancy smoking prevalence (33.2%) than older women (17.6%), overall and in all racial/ethnic groups. Smoking prevalences were higher among younger non-Hispanic whites (46.4%), younger Alaska Natives (55.6%), and younger American Indians (46.9%). After adjusting for confounders, younger non-Hispanic whites, Hispanics, Alaska Natives, and Asian/Pacific Islanders were 1.12 to 1.50 times as likely to smoke as their older counterparts.

**Conclusion:**

Age-appropriate and culturally specific tobacco control interventions should be integrated into reproductive health settings to reach younger non-Hispanic white, Alaska Native, and American Indian women before they become pregnant.

## Introduction

Prenatal smoking remains one of the most common preventable causes of poor pregnancy and infant outcomes (1). Though prenatal smoking prevalence in the United States has declined over time (2), it was 10.4% in 2007, which remains far above the national goals for 2010 and 2020 of 1% (3-5). In addition, prenatal smoking varies greatly among states, from 6.2% in Utah to 35.7% in West Virginia (6).

Although approximately half of female smokers quit when they decide to become pregnant or upon learning that they are pregnant, fewer smokers (5%-12%) quit by the last 3 months of pregnancy (7-9). Even with universal implementation of clinic-based augmented smoking cessation interventions for all pregnant smokers, the overall reduction of the US prenatal smoking prevalence is estimated to be modest, approximately 1.4 percentage points (10). To further reduce prenatal smoking prevalence, tobacco control efforts focused on preventing initiation and increasing smoking cessation before a woman becomes pregnant are needed. This approach would provide smokers with more treatment options, including pharmacotherapy, which is not recommended as a first-line treatment during pregnancy (11). Second, women would have more time to quit smoking, and multiple sessions improve the effectiveness of cessation treatments (12). Finally, targeting women before they become pregnant would reduce the number of women who enter pregnancy smoking, reducing fetal tobacco exposure from the mother.

Our objective was to identify age groups within 6 racial/ethnic groups that have the highest prepregnancy smoking prevalence in a population-based sample of women with a recent live birth. We focused on age and race/ethnicity because they can be used in developing tailored materials. Our study findings will help to identify groups of women most likely to benefit from targeted tobacco control efforts designed to decrease prepregnancy smoking.

## Methods

We analyzed data from the Centers for Disease Control and Prevention (CDC) Pregnancy Risk Assessment Monitoring System (PRAMS) during 2004-2008. PRAMS is an ongoing, population-based surveillance system of maternal behaviors and experiences before, during, and after pregnancy. PRAMS is conducted by state and local health departments in collaboration with CDC. All health departments participating in PRAMS use a standardized data collection methodology developed by CDC (13). At each site, a monthly stratified sample of 100 to 300 new mothers is selected systematically from recent birth certificates. PRAMS staff at each site mail a self-administered questionnaire to the selected women starting 2 to 3 months after the delivery of a live infant. Women who do not respond to any of the 3 serial mailings are contacted by telephone to complete the survey. To minimize recall bias, efforts to contact women end 9 months after the woman has delivered her baby. Survey data are linked to selected birth certificate data and weighted for sample design, nonresponse, and noncoverage. The weighted data represent all live births delivered in each respective site in the given year.

To minimize nonresponse bias, PRAMS sites were included in the analysis if an overall weighted response rate of 70% or more was achieved for 2004 through 2006 and 65% or more for 2007 through 2008 for each site; these thresholds are established by CDC for published results (14). The weighted response rate indicates the proportion of women sampled who completed a survey, adjusting for sample design. Our analysis used data from the following 32 states and New York City for 2004-2008, except where noted: Alaska, Arkansas, Colorado, Delaware (2007-2008), Florida (2004-2005), Georgia, Hawaii, Illinois, Louisiana (2004), Maine, Maryland, Massachusetts (2007-2008), Michigan, Minnesota, Mississippi (2004, 2006, 2008), Missouri (2007), Nebraska, New Jersey, New Mexico (2004-2005), New York (excluding New York City), New York City (2004-2007), North Carolina (2004-2005, 2007-2008), Ohio (2005-2008), Oklahoma, Oregon, Rhode Island, South Carolina (2004-2007), Tennessee (2008), Utah, Washington, West Virginia, Wisconsin (2007-2008), and Wyoming (2007-2008). The PRAMS project has been approved by the CDC institutional review board.

Prepregnancy smoking status was ascertained from the PRAMS questionnaire. Among women who reported smoking in the last 2 years, women were asked how many cigarettes they smoked per day on average during the 3 months before pregnancy. Categorical responses were none (0 cigarettes)Prepregnancy smoking status was ascertained from the PRAMS questionnaire. Among women who reported smoking in the last 2 years, women were asked how many cigarettes they smoked per day on average during the 3 months before pregnancy. Categorical responses were none (0 cigarettes), less than 1, 1 to 5, 6 to 10, 11 to 20, 21 to 40, or 41 or more. Women who reported "none" were classified as nonsmokers; others were classified as prepregnancy smokers. For smokers, number of cigarettes smoked per day on average during the 3 months before pregnancy was collapsed into 3 groups: 1) 5 or fewer cigarettes per day (includes <1 cigarette per day), 2) 6 to 20 cigarettes per day, and 3) more than 20 cigarettes per day.

Maternal demographic characteristics included in the bivariate analysis were age, race/ethnicity, education, Medicaid status (proxy for income), parity, pregnancy intention, state of residence, and year of birth. Age, race/ethnicity, education, parity, state of residence, and year of birth were ascertained from the linked birth certificate data, and Medicaid status and pregnancy intention were ascertained from the PRAMS questionnaire. Maternal age was divided into 2 categories, younger adult (18-24 y) and older adult (≥25 y). We were unable to report analysis of women aged 35 years or older because of inadequate sample size in certain racial/ethnic groups. When we ran adjusted relative risks comparing smoking prevalence of women aged 35 years or older with those aged 20 to 24 years, the conclusions by racial/ethnic group were consistent with our results when we grouped women aged 25 years or older. Maternal race/ethnicity was categorized as non-Hispanic black, non-Hispanic white, Hispanic, Alaska Native, American Indian, and Asian/Pacific Islander. Maternal education was categorized as less than 12 years, 12 years, and greater than 12 years. A woman was classified as enrolled in Medicaid if she reported being on Medicaid just before she got pregnant or if Medicaid was used to pay for prenatal care or for her delivery; otherwise, she was classified as not being enrolled in Medicaid. Parity was categorized as no previous live births or 1 or more previous live births. A pregnancy was categorized as unintended if the mother reported that she had wanted to become pregnant later or not at all.

The analysis was conducted using SAS version 9.2 (SAS Institute, Inc, Cary, North Carolina) and SUDAAN version 10 (Research Triangle Institute, Research Triangle Park, North Carolina), to account for the complex survey design of PRAMS. A total of 200,008 records were available for the analysis among the 32 states and NYC live births during 2004 through 2008; singletons and multiples were included. A woman was excluded if her prepregnancy smoking status (n = 3,444, 1.7%), age (n = 12, <0.1%), or race/ethnicity (n = 1,018, 0.5%) was missing. We excluded women aged less than 18 years (n = 7,287, 3.6%) because we could not adequately control for education, which was reported as a categorical variable. We also excluded women reporting a race other than the 6 specified above or mixed race (n = 2,594, 1.3%). The final number of records analyzed was 186,064. At the time of questionnaire completion, the average infant's age was 120 days and ranged from 61 to 270 days.

We examined the distribution of demographic characteristics for the study population by age group (18-24 y and ≥25 y). We calculated the percentage of unintended pregnancies among smokers and nonsmokers. Next, we calculated prepregnancy smoking prevalence and 95% confidence intervals (CIs) by age group overall and separately for each of the racial/ethnic groups. To explore geographic differences, we examined prepregnancy smoking prevalence by age for each state that had an adequate sample size for each racial/ethnic group. We calculated the proportion of smokers in each of the 3 categories of cigarettes smoked per day. We used χ^2^ tests to examine differences in prevalence estimates by demographic characteristics, state, and the proportion of smokers in each category of cigarettes smoked per day.

For each racial/ethnic group, we modeled the probability of prepregnancy smoking by age group using women aged 25 years or older as the reference population. Unadjusted and adjusted relative risks and 95% CIs were calculated using logistic regression, as described by Bieler et al (15). Maternal education, Medicaid status, parity, pregnancy intention, state of residence, and year of birth were included in the final adjusted models if they confounded the association between age and smoking status by at least 10%.

## Results

Overall, 31.4% of women in our study were aged 18 to 24 years and 68.6% were aged 25 years or older (Table 1). Most women in the study population were non-Hispanic white (62.5%), had more than 12 years of education (54.5%), were not Medicaid insured (54.3%), had previous live births (59.6%), and had intended on getting pregnant (59.7%). The percentage of women by state of residence ranged from 0.2% in Wyoming and Delaware to 9.9% in Illinois, and percentage of women by year of infant birth was 16.8% in 2006 to 22.1% in 2007. The prevalence of unintended pregnancy was higher among smokers (55.4%; 95% CI, 54.6-56.1) than nonsmokers (35.9%; 95% CI, 35.5-36.3) (data not shown).

**Figure 1.**
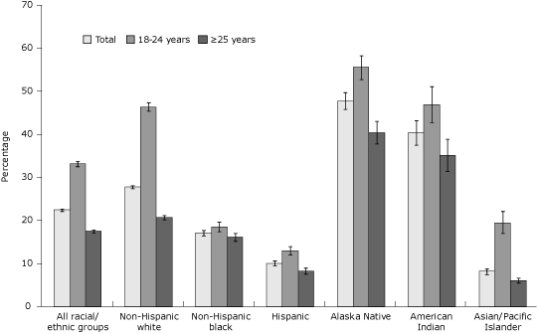
Prepregnancy smoking prevalence by maternal race/ethnicity and age among women who recently delivered a live birth, 32 states and New York City, Pregnancy Risk Assessment Monitoring System, 2004-2008. Prepregnancy smoking prevalence is defined as the percentage of women who recently delivered a live birth who self-reported smoking during the 3 months before pregnancy; error bars represent 95% confidence intervals (CIs) for prepregnancy smoking prevalence. Prepregnancy prevalence comparing women aged 18 to 24 years with women aged 25 years or older was significant (*P* < .05, χ^2^ test) for the overall study population and within all racial/ethnic groups. PRAMS data available for 2004-2008, except where noted: Alaska, Arkansas, Colorado, Delaware (2007-2008), Florida (2004-2005), Georgia, Hawaii, Illinois, Louisiana (2004), Maine, Maryland, Massachusetts (2007-2008), Michigan, Minnesota, Mississippi (2004, 2006, 2008), Missouri (2007), Nebraska, New Jersey, New Mexico (2004-2005), New York (excluding New York City), New York City (2004-2007), North Carolina (2004-2005, 2007-2008), Ohio (2005-2008), Oklahoma, Oregon, Rhode Island, South Carolina (2004-2007), Tennessee (2008), Utah, Washington, West Virginia, Wisconsin (2007-2008), and Wyoming (2007-2008).

For the overall study population, 22.5% of women smoked prepregnancy (Figure 1). Younger women had higher prepregnancy smoking prevalence than older women overall and in all racial/ethnic groups. When examining prevalence by both age and race/ethnicity, different patterns appear. Among women aged 18 to 24 years, Alaska Natives, American Indians, and non-Hispanic whites, estimated  prepregnancy smoking prevalence was higher, whereas it was lower among Hispanics, non-Hispanic blacks, and Asian/Pacific Islanders (Figure 1). Among women aged 25 years or older, Alaska Natives, American Indians, and non-Hispanic whites had higher smoking prevalence estimates while Asian/Pacific Islanders, Hispanics, and non-Hispanic blacks had lower prepregnancy smoking prevalence estimates.

**Table:** 

**Race/ethnicity**	**Total, % (95% CI)**	**18-24 y, % (95% CI)**	**≥25 y, % (95% CI)**
All racial/ethnic groups	22.5 (22.2-22.8)	33.2 (32.6-33.8)	17.6 (17.2-17.9)
Non-Hispanic white	27.8 (27.4-28.2)	46.4 (45.5-47.4)	20.7 (20.3-21.2)
Non-Hispanic black	17.1 (16.5-17.8)	18.5 (17.5-19.7)	16.1 (15.2-17.0)
Hispanic	10.1 (9.6-10.7)	13.0 (12.1-14.0)	8.3 (7.7-9.0)
Alaska Native	47.8 (45.9-49.8)	55.6 (52.8-58.4)	40.4 (37.8-43.1)
American Indian	40.4 (37.6-43.3)	46.9 (42.8-51.2)	35.2 (31.5-39.0)
Asian/Pacific Islander	8.2 (7.5-8.9)	19.5 (17.0-22.2)	6.1 (5.5-6.8)

For almost all study sites, prepregnancy smoking prevalence was higher for younger women than for older women for each racial/ethnic group; not all estimates were significantly different (*P* < .05, χ^2^ test). For non-Hispanic whites, prepregnancy smoking in young women was significantly higher than for older women across all study states except New York City, which did not have an adequate sample size.

**Figure 2.**
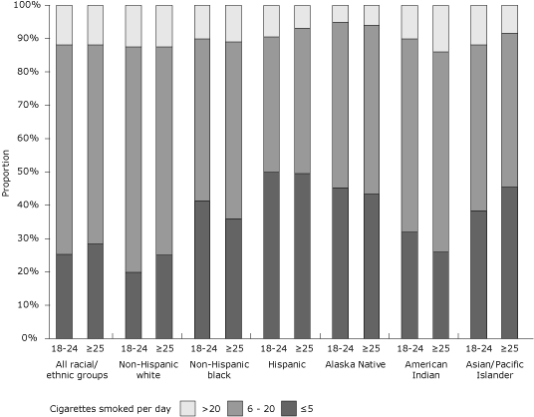
Proportion of prepregnancy smokers by average number of cigarettes smoked per day, by maternal race/ethnicity and age among women who recently delivered a live birth, 32 states and New York City, Pregnancy Risk Assessment Monitoring System (PRAMS), 2004-2008. Prepregnancy smoking is defined as self-reported smoking of any amount of cigarettes during the 3 months before pregnancy. Proportion of prepregnancy smokers by average number of cigarettes smoked per day was significant (*P* < .05, χ^2^ test) comparing women aged 18 to 24 years with women aged 25 years or older for the overall study population and among non-Hispanic whites. PRAMS data available for 2004-2008, except where noted: Alaska, Arkansas, Colorado, Delaware (2007-2008), Florida (2004-2005), Georgia, Hawaii, Illinois, Louisiana (2004), Maine, Maryland, Massachusetts (2007-2008), Michigan, Minnesota, Mississippi (2004, 2006, 2008), Missouri (2007), Nebraska, New Jersey, New Mexico (2004-2005), New York (excluding New York City), New York City (2004-2007), North Carolina (2004-2005, 2007-2008), Ohio (2005-2008), Oklahoma, Oregon, Rhode Island, South Carolina (2004-2007), Tennessee (2008), Utah, Washington, West Virginia, Wisconsin (2007-2008), and Wyoming (2007-2008).

Among prepregnancy smokers overall, 26.9% smoked 5 or fewer cigarettes per day, 61.2% smoked 6 to 20 cigarettes per day, and 11.9% smoked more than 20 cigarettes per day. A significant difference was seen only in non-Hispanic whites (*P* < .05, χ^2^ test); the proportion of younger white smokers who smoked 6 to 20 cigarettes per day (67.8%; 95% CI, 66.5-69.1) was higher than the proportion of older white smokers who smoked at the same levels (62.6%; 95% CI, 61.4-63.8) (Figure 2). The percentage of smokers who smoked less than 1 cigarette per day was low in all racial/ethnic groups (range, 2.4% in blacks to 8.7% in Hispanics).

**Table:** 

**Race/ethnicity and Age, y**	**Average No. of Cigarettes Smoked Per Day**		

	**≤5**	**6-20**	**>20**
**All racial/ethnic groups, y**			
18-24	25.2	62.9	11.9
≥25	28.4	59.8	11.9
**Non-Hispanic white, y**			
18-24	19.7	67.8	12.5
≥25	25.0	62.6	12.5
**Non-Hispanic black, y**			
18-24	41.2	48.6	10.2
≥25	35.9	53.0	11.1
**Hispanic, y**			
18-24	49.8	40.6	9.6
≥25	49.4	43.6	7.0
**Alaska Native, y**			
18-24	45.1	49.7	5.1
≥25	43.3	50.6	6.1
**American Indian, y**			
18-24	31.8	58.0	10.2
≥25	25.8	60.2	13.9
**Asian/Pacific Islander, y**			
18-24	38.3	49.7	12.0
≥25	45.3	46.2	8.5

After adjusting for maternal education, Medicaid enrollment, parity, pregnancy intention, state of residence, and year of birth, the associations between prepregnancy smoking and maternal age for non-Hispanic white, Hispanic, Alaska Native, and Asian/Pacific Islander women remained significant (Table 2). Compared to their older counterparts, younger Asian/Pacific Islanders, younger Hispanics, younger Alaska Natives, and younger non-Hispanic whites were more likely to smoke prepregnancy. Compared with their older counterparts, younger non-Hispanic blacks were less likely to smoke prepregnancy. After controlling for confounders, the association between prepregnancy smoking and age was not significant for American Indians.

## Discussion

We found that approximately 1 of 2 women aged 18 to 24 years of non-Hispanic white, American Indian, or Alaska Native race/ethnicity smoked prepregnancy. Overall by age, we found that prepregnancy smoking prevalence was higher in younger than in  older women. This estimate is higher than the current smoking prevalence among nonpregnant women (22%) obtained from the Behavioral Risk Factor Surveillance System (16). In addition, in our study, compared with their older counterparts, younger women with a recent live birth were more likely to have other risk factors for poor birth outcomes, such as being less educated and more likely to be enrolled in Medicaid (a proxy for lower income), and younger non-Hispanic white women were more likely to be heavier smokers.

Together, these findings suggest that young women who become pregnant, specifically non-Hispanic whites, American Indians, and Alaska Natives, are more likely to be smokers than those who do not become pregnant. For non-Hispanic whites, we observed these differences by age across all states. These disparities may be due to a host of factors, such as social context, cultural influences, and biological differences in nicotine addiction (17). Because the tobacco industry is banned from marketing to minors, it is shifting its marketing toward young adults through popular media outlets and venues frequented by young adults (18). Results from a national survey indicated that smoking in young adults has been linked to receptivity to tobacco advertising and being exposed to advertising (19). Further research is needed to better understand the profile of young female smokers so that tobacco control programs can effectively reach them.

Smoking cessation services should be integrated into health care settings that young women at risk for pregnancy are likely to attend, such as family planning clinics. We found that a greater percentage of smokers than nonsmokers reported that their recent live birth was unintended, suggesting a need for appropriate contraceptive counseling in addition to cessation services. Providers should ask all women about smoking, and cessation should be encouraged before pregnancy, when the most treatment options, including pharmacotherapy, are available. Services and materials are more effective when they are made age-appropriate and culturally, racially, and educationally appropriate for the patient (12). Also, telephone-based quitlines have been found to be effective in reaching diverse populations (12). Each state has a telephone quitline that provides free cessation counseling, and often additional services, to all smokers. Because young women are less likely to seek general preventive care (20), integrating chronic disease prevention into routine reproductive health visits, such as contraceptive visits, may help reduce smoking in these at-risk women.

Our study has several limitations. First, smoking status was self-reported and not biochemically validated. In a recent study using biochemical validation, the authors found that pregnant smokers were less likely to disclose their current smoking status than nonpregnant women (21). However, it is unclear to what extent prepregnancy smoking is underreported among women with a recent live birth. Second, the number of cigarettes smoked per day in the 3 months before pregnancy was reported by the mothers an average of 120 days after delivery and so may be subject to recall bias. Third, we excluded pregnancies among women aged less than 18 years from our analysis because we were unable to adequately control for education. These young women are at high risk for unintended pregnancies, and further research is needed to better understand prepregnancy smoking patterns in this population. Lastly, our findings are generalizable only to women who delivered a recent live birth in the study states and given study year, and so are not generalizable to the entire United States or to women whose pregnancy does not end in live birth, such as stillbirths or miscarriages. Despite these limitations, this is one of the first population-based studies examining disparities in prepregnancy smoking among a representative sample of women who delivered live births.

In summary, we found significant disparities in prepregnancy smoking among younger women and within racial/ethnic groups. To reach these women with higher rates of smoking, evidence-based interventions should be tailored for these populations and should educate them on the harms of smoking if they become pregnant. In addition, chronic-disease prevention programs should be integrated into reproductive health clinical settings, which women are most likely to attend. Effective tobacco control interventions in younger women will lead to improved overall maternal health and the prevention of poor pregnancy outcomes.

## Figures and Tables

**Table 1:** Demographic Characteristics by Maternal Age Among Women Who Recently Delivered a Live Birth, 32 States and New York City,^a^ Pregnancy Risk Assessment Monitoring System (PRAMS), 2004-2008

**Characteristic**	Total, % (95% CI) (Unweighted n = 186,064)	18-24 y, % (95% CI) (Unweighted n = 61,759)	≥25 y, % (95% CI) (Unweighted n = 124,305)	*P* Value^b^
**Total^c^**	100.0	31.4 (31.0-31.7)	68.6 (68.3-69.0)	NA
**Race/ethnicity**				
Non-Hispanic white	62.5 (62.2-62.8)	54.9 (54.2-55.5)	66.0 (65.6-66.4)	<.01
Non-Hispanic black	15.4 (15.2-15.7)	21.5 (21.0-22.0)	12.7 (12.4-12.9)	
Hispanic	16.3 (16.0-16.5)	19.9 (19.3-20.4)	14.6 (14.3-15.0)	
Alaska Native	0.1 (0.1-0.1)	0.2 (0.2-0.2)	0.1 (0.1-0.1)	
American Indian	0.9 (0.8-0.9)	1.2 (1.1-1.3)	0.7 (0.6-0.8)	
Asian/Pacific Islander	4.8 (4.6-4.9)	2.3 (2.2-2.5)	5.9 (5.7-6.0)	
**Education, y**				
<12	16.0 (15.7-16.2)	26.9 (26.3-27.5)	11.0 (10.7-11.3)	<.01
12	29.5 (29.2-29.8)	44.7 (44.1-45.4)	22.5 (22.2-22.9)	
>12	54.5 (54.2-54.9)	28.4 (27.8-29.0)	66.5 (66.1-66.9)	
**Medicaid enrollment^d^**				
Yes	45.7 (45.3-46.0)	74.1 (73.5-74.6)	32.7 (32.3-33.1)	<.01
No	54.3 (54.0-54.7)	25.9 (25.4-26.5)	67.3 (66.9-67.7)	
**Parity**				
0	40.4 (40.0-40.7)	56.9 (56.3-57.6)	32.8 (32.4-33.2)	<.01
≥1	59.6 (59.3-60.0)	43.1 (42.4-43.7)	67.2 (66.8-67.6)	
**Pregnancy intention**				
Intended	59.7 (59.4-60.1)	40.8 (40.2-41.5)	68.4 (68.0-68.8)	<.01
Unintended	40.3 (39.9-40.6)	59.2 (58.5-59.8)	31.6 (31.2-32.0)	
**State of residence**				
Alaska	0.6 (0.6-0.6)	0.7 (0.7-0.7)	0.6 (0.5-0.6)	<.01
Arkansas	2.1 (2.1-2.1)	3.0 (2.9-3.1)	1.7 (1.7-1.7)	
Colorado	3.9 (3.9-4.0)	3.7 (3.5-3.9)	4.1 (4.0-4.1)	
Delaware	0.2 (0.2-0.2)	0.2 (0.2-0.2)	0.2 (0.2-0.2)	
Florida	4.9 (4.9-5.0)	5.6 (5.3-5.9)	4.6 (4.5-4.7)	
Georgia	7.7 (7.6-7.8)	9.0 (8.5-9.4)	7.2 (7.0-7.4)	
Hawaii	1.1 (1.1-1.1)	1.1 (1.0-1.1)	1.1 (1.1-1.1)	
Illinois	9.9 (9.8-9.9)	9.0 (8.7-9.4)	10.2 (10.1-10.4)	
Louisiana	0.7 (0.7-0.7)	1.0 (0.9-1.1)	0.6 (0.6-0.6)	
Maine	0.8 (0.8-0.8)	0.8 (0.8-0.8)	0.8 (0.8-0.8)	
Maryland	3.8 (3.7-3.8)	3.3 (3.1-3.5)	4.0 (3.9-4.1)	
Massachusetts	1.7 (1.7-1.7)	1.1 (1.0-1.2)	2.0 (1.9-2.0)	
Michigan	6.6 (6.5-6.6)	6.7 (6.4-7.0)	6.5 (6.4-6.7)	
Minnesota	3.8 (3.8-3.9)	3.1 (2.9-3.2)	4.2 (4.1-4.2)	
Mississippi	1.2 (1.2-1.2)	1.8 (1.8-1.9)	1.0 (0.9-1.0)	
Missouri	0.9 (0.9-0.9)	1.0 (1.0-1.1)	0.8 (0.8-0.9)	
Nebraska	1.5 (1.5-1.5)	1.4 (1.4-1.5)	1.5 (1.5-1.5)	
New Jersey	6.1 (6.1-6.2)	4.2 (4.0-4.4)	7.0 (6.9-7.1)	
New Mexico	0.5 (0.5-0.5)	0.7 (0.7-0.7)	0.4 (0.4-0.4)	
New York	6.1 (6.1-6.2)	4.6 (4.3-4.9)	6.8 (6.7-6.9)	
New York City	4.4 (4.3-4.4)	3.8 (3.5-4.0)	4.6 (4.5-4.7)	
North Carolina	5.2 (5.1-5.2)	5.7 (5.4-5.9)	5.0 (4.9-5.1)	
Ohio	6.4 (6.4-6.5)	6.9 (6.6-7.3)	6.2 (6.1-6.4)	
Oklahoma	2.9 (2.9-2.9)	3.9 (3.8-4.1)	2.5 (2.4-2.5)	
Oregon	2.6 (2.6-2.7)	2.6 (2.5-2.8)	2.7 (2.6-2.7)	
Rhode Island	0.7 (0.7-0.7)	0.6 (0.6-0.6)	0.7 (0.7-0.7)	
South Carolina	2.2 (2.1-2.2)	2.7 (2.6-2.9)	1.9 (1.9-2.0)	
Tennessee	0.9 (0.9-1.0)	1.1 (1.0-1.3)	0.8 (0.8-0.9)	
Utah	3.0 (3.0-3.1)	3.2 (3.1-3.3)	3.0 (2.9-3.0)	
Washington	4.7 (4.6-4.7)	4.4 (4.2-4.6)	4.8 (4.7-4.9)	
West Virginia	1.0 (0.9-1.0)	1.3 (1.2-1.4)	0.8 (0.8-0.8)	
Wisconsin	1.6 (1.6-1.6)	1.5 (1.4-1.6)	1.7 (1.6-1.7)	
Wyoming	0.2 (0.2-0.2)	0.2 (0.2-0.2)	0.2 (0.1-0.2)	
**Year of birth**				
2004	20.2 (20.1-20.3)	20.8 (20.4-21.3)	19.9 (19.7-20.1)	.013
2005	20.6 (20.5-20.6)	20.8 (20.4-21.3)	20.4 (20.2-20.6)	
2006	16.8 (16.7-16.9)	16.7 (16.3-17.1)	16.8 (16.7-17.0)	
2007	22.1 (22.0-22.2)	21.8 (21.3-22.3)	22.3 (22.1-22.5)	
2008	20.3 (20.2-20.4)	19.8 (19.4-20.3)	20.5 (20.3-20.7)	

Abbreviation: CI, confidence interval; NA, not applicable.

a PRAMS data available for 2004-2008, except where noted: Alaska, Arkansas, Colorado, Delaware (2007-2008), Florida (2004-2005), Georgia, Hawaii, Illinois, Louisiana (2004), Maine, Maryland, Massachusetts (2007-2008), Michigan, Minnesota, Mississippi (2004, 2006, 2008), Missouri (2007), Nebraska, New Jersey, New Mexico (2004-2005), New York (excluding New York City), New York City (2004-2007), North Carolina (2004-2005, 2007-2008), Ohio (2005-2008), Oklahoma, Oregon, Rhode Island, South Carolina (2004-2007), Tennessee (2008), Utah, Washington, West Virginia, Wisconsin (2007-2008), and Wyoming (2007-2008).

b χ^2^ test.

c Row percents provided.

d Medicaid enrollment is defined as report of being on Medicaid just before pregnancy or Medicaid was used to pay for prenatal care or for delivery.

**Table 2:** Relative Risk of Prepregnancy Smoking^a^ by Maternal Age Within Racial/Ethnic Groups Among Women Who Recently Delivered a Live Birth, 32 States and New York City,^b^ Pregnancy Risk Assessment Monitoring System (PRAMS), 2004-2008

**Maternal Race/Ethnicity and Age**	Unadjusted RR (95% CI)	Adjusted RR^c^ (95% CI)
**Non-Hispanic white** (unweighted n = 102,870), y		
18-24	2.24 (2.18-2.31)	1.12 (1.08-1.16)
≥25	Reference	Reference
**Non-Hispanic black** (unweighted n = 31,802), y		
18-24	1.15 (1.07-1.25)	0.90 (0.82-0.98)
≥25	Reference	Reference
**Hispanic** (unweighted n = 28,119), y		
18-24	1.56 (1.40-1.74)	1.41 (1.25-1.60)
≥25	Reference	Reference
**Alaska Native** (unweighted n = 2,297), y		
18-24	1.38 (1.27-1.49)	1.20 (1.09-1.32)
≥25	Reference	Reference
**American Indian** (unweighted n = 5,044), y		
18-24	1.33 (1.16-1.53)	1.04 (0.90-1.20)
≥25	Reference	Reference
**Asian/Pacific Islander** (unweighted n = 15,932), y		
18-24	3.19 (2.67-3.80)	1.50 (1.19-1.88)
≥25	Reference	Reference

Abbreviations: RR, relative risk; CI, confidence interval.

a Prepregnancy smoking is defined as self-reported smoking of any amount of cigarettes during the 3 months before pregnancy.

b PRAMS data available for 2004-2008, except where noted: Alaska, Arkansas, Colorado, Delaware (2007-2008), Florida (2004-2005), Georgia, Hawaii, Illinois, Louisiana (2004), Maine, Maryland, Massachusetts (2007-2008), Michigan, Minnesota, Mississippi (2004, 2006, 2008), Missouri (2007), Nebraska, New Jersey, New Mexico (2004-2005), New York (excluding New York City), New York City (2004-2007), North Carolina (2004-2005, 2007-2008), Ohio (2005-2008), Oklahoma, Oregon, Rhode Island, South Carolina (2004-2007), Tennessee (2008), Utah, Washington, West Virginia, Wisconsin (2007-2008), and Wyoming (2007-2008).

c Adjusted for maternal education, Medicaid enrollment, parity, pregnancy intention, state of residence, and year of birth.
